# Tubulin structure-based drug design for the development of novel 4β-sulfur-substituted podophyllum tubulin inhibitors with anti-tumor activity

**DOI:** 10.1038/srep10172

**Published:** 2015-05-11

**Authors:** Wei Zhao, Jia-Ke Bai, Hong-Mei Li, Tao Chen, Ya-Jie Tang

**Affiliations:** 1 Key Laboratory of Fermentation Engineering (Ministry of Education), Hubei Provincial Cooperative Innovation Center of Industrial Fermentation, Hubei University of Technology, Wuhan 430068 China; 2 Key Laboratory of Systems Bioengineering (Ministry of Education), School of Chemical Engineering and Technology, Tianjin University, Tianjin 300072 China

## Abstract

The well-characterized anti-tubulin agent, podophyllotoxin (PTOX), with the 4′-position methoxyl group, targets the colchicines domain located between α- and β-tubulin. Two guanosine triphosphate (GTP) analogs of the tubulin-binding region were synthesized from PTOX, where a hydroxyl group was substituted with a carbon-sulfur bond. These compounds, 4-MP-PTOX and 4-TG-PTOX, reduce the dosage and greatly improve the therapeutic effect for microtubule damage in cancer cells. Here we characterize the anti-tubulin properties of these compounds. We found the stronger inhibition of tubulin polymerization (the concentration of 50% growth inhibition, GI50 < 2 μM) for compounds 4-TG-PTOX and 4-MP-PTOX, which were better than that of PTOX or colchicine. The cytotoxicity of two designed compounds on tumor cells was also significantly enhanced by comparing to those of PTOX and colchicines. The Δ*H* value of 4-MP-PTOX and 4-TG-PTOX binding to tubulin by isothermal titration calorimetry (ITC) was found to be −7.4 and −5.3 kcal·mol^−1^, respectively. The wide range of enthalpy values across the series may reflect entropy/enthalpy compensation effects. Fragments 6-mercaptopurine (MP) and 6-thioguanine (TG) likely enhance the affinity of 4-MP-PTOX and 4-TG-PTOX binding to tubulin by increasing the number of binding sites. The correctness of rational drug design was strictly demonstrated by a bioactivity test.

Tubulin is a key component of the cytoskeletal network and is important in a wide range of cellular functions. It is particularly critical in the cell life cycle^1^, and blocking tubulin polymerization causes cells in metaphase to arrest mitosis. Since cancer cells undergo uncontrolled, abnormal mitosis, tubulin is an attractive molecular target for novel cancer therapies^2^,^3^.

Podophyllotoxin (PTOX) is one of the most well-known naturally-occurring aryltetralin lignans exhibiting anti-tumor activity^4^,^5^. PTOX binds at the interface between α- and β-tubulin, which inhibits the assembly of tubulin into microtubules. The trimethoxyphenyl (TMP) group of podophyllotoxin derivatives has been shown to bind via hydrophobic interactions with Leu, Ala, and Cys residues of the β-tubulin interface^4^. An effort is underway to use podophyllum compounds as the model for natural lead compounds, and to develop a tubulin-targeting inhibition strategy for novel anti-tumor drugs.

Bioisosteres are substituents with similar physical or chemical properties that produce broadly similar biological properties to a chemical compound. They are used to improve drug activity levels and to reduce the toxicity of the lead compound^6^. For example, both the oxygen and sulfur atoms have the same number of valence electrons, but the electron cloud of the sulfur atom, with greater electronegativity, is denser than that of the oxygen atom. Thus, the sulfur atom preferentially integrates with the protein molecule with the hydrogen bond in the tumor cell. Our previous work demonstrated that sulfur substitution improves the anti-tumor activity of PTOX^7^. And tubulin-binding groups substitute the hydroxyl group with a carbon-sulfur bond at the cycloparaffin (C-ring) 4-position (C-4) of PTOX was a good modification direction. This modification reduces the dosage and greatly improves the therapeutic effects on microtubule damage in cancer cells by inhibiting tubulin polymerization.

6-mercaptopurine (6-MP)^8^ and 6-thioguanine (6-TG)^9^ are analogs of guanosine triphosphate (GTP), which is essential for tubulin polymerization. GTP and its analogs have a strong affinity for the Asn residue at the β-tubulin interface which is the growing end of microtubules^10^. These GTP analogs are sulfur-containing heterocyclic compounds with higher electron densities surrounding the sulfur atom and therefore have a higher affinity for tubulin. For this reason, 6-MP and 6-TG are good functional models for the substitution for a carbon-sulfur bond at the C-4 position of PTOX to enhance the stability of tubulin polymerization.

This work provides a potential route in the development of the novel podophyllum tubulin inhibitors for anti-tumor treatment. We describe an important class of anti-tumor agents, their determinants on tubulin binding affinity, and pave the way for further investigation into the efficacy of these drugs anti-tumor agents.

## Results and Discussion

**Figure 1:**
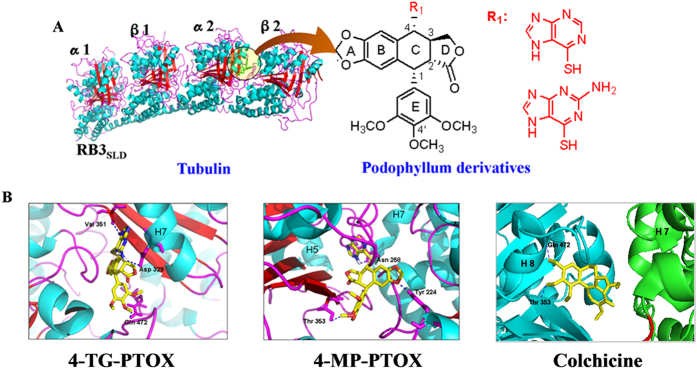
**A**. Structure of the tubulin cleavage complex stabilized by the compounds. **B**. The 4β-sulfur-substituted podophyllum derivatives and colchicines binding site on tubulin and detailed structures of the compounds-binding sites. The blue dotted lines are H-bonds.

In this study, we utilized a tubulin stathmin-like domain complex (Brookhaven Protein Data Bank; PDB code 1SA1^10^) as the target structure in molecular docking. The asymmetric unit was composed of compounds symmetrically enclosed by tubulin (Fig. 1A). The phenyl ring of the compounds was located at the α/β interface of tubulin. Abad et al. (2012) reported that the lipophilic nature of the tubulin region around the trimethoxyphenyl fragment (β-unit of tubulin) could be observed, and the hydrogen bonds with the hydroxyl group of threonine (α-unit of tubulin) involved in the pinacol function are also present^11^. The root-mean-square deviation (RMSD) was calculated for each configuration and compared with the co-crystallized compounds. This value was found to be between 0.04 and 2.74 Å. The RMSD value calculated from the accepted positions for each configuration was found to be between 0.62 and 1.52 Å. The best docked structures, which were those that exhibited configurations with the lowest glide scores, were compared with their crystal structures (Fig. 1B). Analysis of the docking studies revealed that the trimethoxyphenyl (TMP) group of 4β-S-(6-mercaptopurine-yl)sulfanyl-4-deoxy-podophyllotoxin (4-MP-PTOX), 4β-S-(6-thioguanine-yl)sulfanyl-4-deoxy-podophyllotoxin (4-TG-PTOX), and colchicine could bind via hydrophobic interactions with the β-unit of tubulin at residues Gln 472 and Thr 353, respectively. The phenyl ring of the compounds was located at the α/β interface of tubulin. Furthermore, Compared with colchicine, the 6-mercaptopurine (6-MP) and 6-thioguanine (6-TG) in the 4-MP-PTOX and 4-TG-PTOX compounds served as hydrogen bond acceptors to Val 351, Asn 258 and Asp 329 of the main chain. All of the ligands bound to tubulin and fit well into the defined binding pocket. The binding energies of 4-MP-PTOX and 4-TG-PTOX were found to be −9.75 kcal/mol and −11.63 kcal/mol respectively, which were better than that of the parent compound, PTOX (interaction energy −7.52 kcal/mol) and the reference compound colchicine (interaction energy −9.62 kcal/mol). We therefore investigated whether 4-MP-PTOX and 4-TG-PTOX could also act as tubulin inhibitors, which have been shown to act as potential anti-tumor agents. *In silico* predictions correlated well with data obtained from our *in vitro* cytotoxicity assays. We report here on the anti-tubulin properties of these two novel 4β-sulfur-substituted podophyllum derivatives, which were designed by the structure-based dual-target design strategy based on the structure of tubulin.

**Figure 2:**
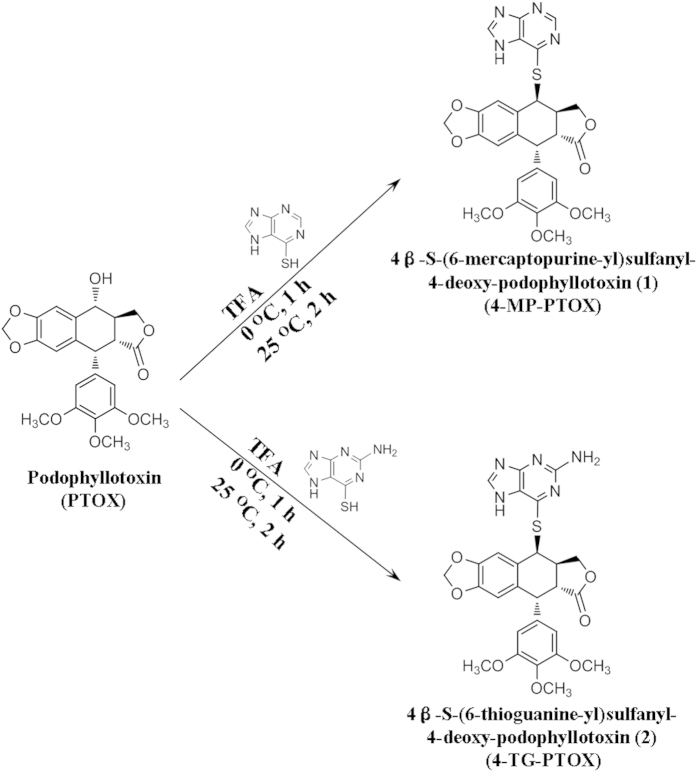
Synthesis of 4β-sulfur-substituted podophyllum derivatives from PTOX in the presence of TFA.

**Figure 3:**
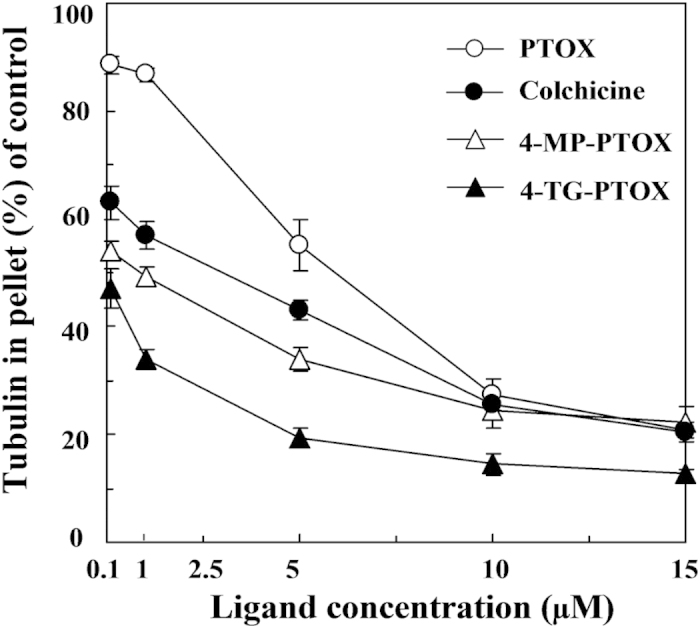
Inhibition of tubulin assembly *in vitro* by podophyllotoxin (PTOX), colchicines, 4-MP-PTOX, or 4-TG-PTOX.

**Figure 4:**
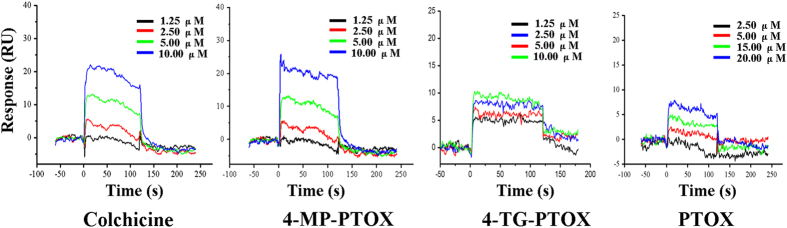
SPR measurements of binding kinetics of colchicines, 4-MP-PTOX, 4-TG-PTOX, and PTOX to tubulin.

First, the starting substrate of PTOX was modified via 4β-sulfur-substitution to produce two novel compounds, namely 4-MP-PTOX and 4-TG-PTOX (Fig. 2). The 4-hydroxyl position of PTOX was first 4β-sulfur-substituted by 6-MP and then 6-TG to synthesize the 4β-sulfur-substituted podophyllum derivatives, 4-MP-PTOX and 4-TG-PTOX, in the presence of TFA. The spectrum of NMR (Fig. 1S, 2S) and FT-IR (Fig. 3S) was shown in method section and supplementary material. The spectrum of the additional peak at Rt = 37.8 (Fig. 4SA) and 24.3 (Fig. 4SB) min showed the protonated ion at *m⁄z* = 549.0 ([M_1_ + H]^+^), 564.1 ([M_2_ + H]^+^), 535.0 ([M_3_ + H]^+^), and 550.1 ([M_4_ + H]^+^), respectively; this observation was consistent with a 4-MP-PTOX molecular weight (M_*W*_) of 548 and a 4-TG-PTOX M_*W*_ of 563. Meanwhile, main fragment ions were observed at *m⁄z* = 152.9 (Fig. 4SA) and 167.9 (Fig. 4SB); this observation was consistent with a 6-MP M_*W*_ of 152 and a 6-TG M_*W*_ of 167. To assess the validity of the structure-function relationship predicted *in silico*, the biological activities of these two derivatives were evaluated by assays analyzing microtubule polymerization and depolymerization, tubulin binding affinity, cell cycle arrest, induction of apoptosis, and cytotoxicity.

**Figure 5:**
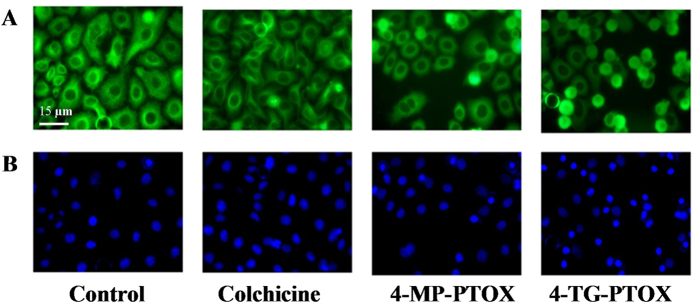
Colchicines, 4-MP-PTOX, and 4-TG-PTOX inhibited tubulin polymerization in A549 cells. **A**. The morphology of microtubules (green). **B**. The morphology of nuclei (blue).

The degree of tubulin polymerization was evaluated through pellet mass formation in centrifugation assays in the presence of stoichiometric and semi-stoichiometric concentrations of each lignan. Inhibition curves were used to determine GI_50_, which is the concentration that causes 50% growth inhibition. We found stronger inhibition (GI_50_ < 1 μM) for compound 4-TG-PTOX (Fig. 3). To further investigate the binding affinity of 4-MP-PTOX and 4-TG-PTOX to tubulin, surface plasmon reonance (SPR) was employed. As shown in Fig. 4, the response unit (RU) increased in an inhibitor concentration-dependent manner. The equilibrium dissociation constant (*K*_D_ value) of 4-MP-PTOX (8.7 μM) and 4-TG-PTOX (6.2 μM) were approximately 1.5 × lower than that of PTOX (13.7 μM). Further, the *K*_D_ for 4-TG-PTOX was slightly lower than that of colchicines (8.5 μM). These data indicate that 6-MP and 6-TG, with the higher electron density around the sulfur atom, could facilitate the stabilization of PTOX-binding to tubulin. Next, we found that the microtubule polymerization and spindle formation were significantly impaired by treatment with 4-MP-PTOX and 4-TG-PTOX (Fig. 5A). In these experiments, A549 cells were treated with either 4-MP-PTOX (1 μM) or 4-TG-PTOX (1 μM) for 12 hours, and anti-microtubule activity was observed for both compounds. Some cells were micronucleated, and some cells arrested during the mitotic phase with a bundle of condensed DNA and no mitotic spindle (Fig. 5A). The nucleus was not damaged by either 4-MP-PTOX and 4-TG-PTOX (Fig. 5B). These results indicated that the 4-MP-PTOX and 4-TG-PTOX were able to depolymerize cellular microtubules.

**Figure 6:**
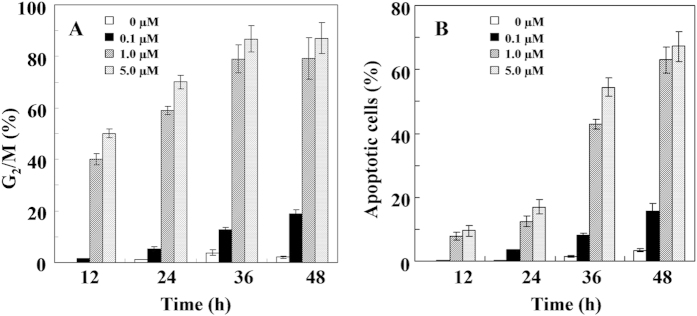
Effects of 4-TG-PTOX on the A549 cell cycle arrest (**A**) and apoptosis-inducing (**B**).

Tubulin inhibitors block cell cycle progression in mitosis at the G_2_/M phase, and a prolonged mitotic arrest triggers various apoptotic pathways^12^. In the presence of low concentrations (0.1 μM) of 4-TG-PTOX, we found that the G_2_/M phase was arrested after 12 h and strongly arrested after 24 and 48 h of incubation and under higher concentrations (1 and 5 μM; Fig. 6A). At 4-TG-PTOX concentrations between 1 and 5 μM, the G_2_/M phase arrest ratio rapidly increased after 6-24 h. After 24 h incubations, the G_2_/M phase arrest ratio remained at around 80% and 90% until the end of the incubation. No significant G_2_/M phase arrest was observed in the absence of 4-TG-PTOX. Together, these results indicate that 4-TG-PTOX causes a complete depolymerization of the microtubule cytoskeleton. This is in line with previous reports that PTOX with the para-position methoxyl group (4′-MeO) is a mitotic spindle agent that inhibits the polymerization of tubulin and stops cell division at the G_2_/M phase^13^.

We also found that 4-TG-PTOX significantly induced apoptosis at various concentrations (0.1, 1, and 5 μM). The ratio of apoptotic cells was significantly elevated by the higher concentrations of 1 and 5 μM, but was unaffected by the lower concentration of 0.1 μM (Fig. 6B). Temporally, apoptosis levels initially remained low at concentrations of 1 and 5 μM, but rapidly increased after 12-24 h. After 48 h of treatment with 4-TG-PTOX, the maximum levels of apoptosis reached were 63% and 67% at 4-TG-PTOX concentrations of 1 and 5 µM, respectively. No significant amount of apoptosis was observed in the absence of 4-TG-PTOX. These data indicate that 4-TG-PTOX could lead to a complete depolymerization of the microtubule cytoskeleton. Importantly, Fig. 6 clearly show that the G_2_/M phase arrest ratio quickly increased after 12 to 36 h of treatment with 1 and 5 μM of 4-TG-PTOX, but a lag phase of 12 hours was observed in cell apoptosis-inducing treatment with both 1 and 5 μM 4-TG-PTOX. Together, these data demonstrate that an increase in the total amount of DNA might be the result of mitotic arrest in A549 cells treatment with 4-TG-PTOX, thereby inducing apoptosis.

As displayed in Table 1, the anti-tumor activities of 4-MP-PTOX (IC_50_ values of 1.12 ± 0.35 μM) and 4-TG-PTOX (IC_50_ values of 0.57 ± 0.92 μM) against the tumor cell line A549 were 17 times and 34 times better than that of PTOX (IC_50_ values of 20.15 ± 2.75 μM), respecively. Further, they were 13 times and 27 times better than that of the well-known tubulin inhibitor colchicines (IC_50_ values of 12.23 ± 0.76 μM) and 24 times and 47 times better than that of the best-known microtubule depolymerizing agent and the clinically important anticancer drug, nocodazole (IC_50_ of 27.44 ± 2.92 μM), respectively. The IC_50_ values indicated that our two compounds have promising anti-tumor activity and less cytotoxicity in the human cell line HL-7720, which were better than the standard compounds (e.g. PTOX or nocodazole) (Table 1). It should be noted that both the sulfur atom and the oxygen atom have the same number of valence electrons, though the electron cloud of the sulfur atom, with higher electronegativity, is denser than that of the oxygen atom. Thus, the sulfur atom preferentially integrated with the large proteins via hydrogen bonds in tumor cells, and sulfur substitution is an effective modification resulting in improved anti-tumor activity. Furthermore, the IC_50_ value of 4-MP-PTOX (11.30 ± 0.57 μM) and 4-TG-PTOX (13.51 ± 2.41 μM) in the human proliferating phenotypes cell line HL-7720 was approximately 2.6 times and 3 times higher than that of PTOX (4.31 ± 1.46 μM), respectively. Further, they were approximately 1.7 times and 2 times higher than that of colchicines (6.42 ± 1.03 μM) and approximately 1.3 times and 1.6 times higher than that of nocodazole (8.51 ± 1.73 μM), respectively. These results indicate that the substitution of the sulfur-containing heterocyclic compounds with a 4β-configuration at position 4 of PTOX is a very useful modification, resulting in enhanced anti-tumor activity and reduced toxic side-effects. The 4β-sulfur-substituted 6-MP or 6-TG with a C-S bond at position 4 of PTOX is thus responsible for the observed anti-tumor activity improvement and for decreasing toxic side-effects. Furthermore, it was noted that some of the IC_50_ standard deviations were extraordinarily large, regularly more than 10% of the reported value, especially, the lowest IC_50_ value of 4-TG-PTOX on the A549 and HepG2 cells. The tumor inhibition experiment was strictly repeated six times and the result was real and valid. From the physical and chemical properties views, almost all of podophyllum compounds suffer from poor water solubility due to the multi-aromatic rings and phenylpropanoid structure. So, this poor water solubility could direct effects on the cellular uptake rate or quantitative analysis result in the significant variability of the IC_50_ standard deviations. Actually, the phenomenon of the IC_50_ standard deviations large more than 10% generally existed in other antitumor studies^15^,^16^,^17^,^18^. In our further drug design study of novel podophyllum antitumor compounds, the poor water solubility issues need to be improved by adding other group with pharmacokinetic properties.

It is estimated that the proliferation rate of most of tumor cells are nearly 10-50 × faster than that of normal cells and must circumvent powerful programs that negatively regulate cell proliferation^14^. Tubulin is involved in a wide range of cellular functions and is essential for a healthy cell cycle. The inhibition of tubulin polymerization causes cells in metaphase to arrest. Cell cycle checkpoints act to protect cells from external stresses and internal errors that would compromise the integrity of the cell. Checkpoints are often defective in tumor cells. Drugs targeting tubulin and thus cell cycle checkpoints could provide selectivity and cytotoxicity of tumor cells. In this work, two tubulin polymerization inhibitors, 4-MP-PTOX and 4-TG-PTOX, strongly arrested the tumor cells at the G_2_/M phase. The compounds may also trigger a G_2_-phase checkpoint response in normal cells, but are only cytotoxic in tumor cells, where this checkpoint is defective. This notion is supported by the study of Warrener *et al.* (2013) who showed that the tumor-selective cytotoxicity of these drugs caused by the disruption of two cell cycle checkpoints^19^.

**Figure 7:**
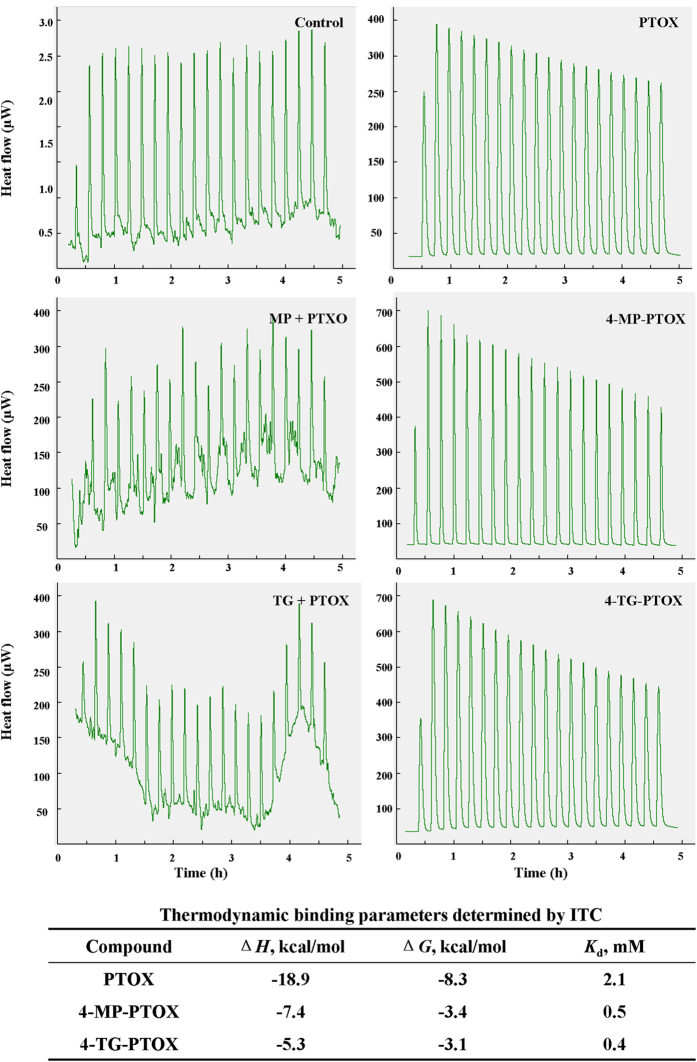
Binding isotherms for 4-MP-PTOX and 4-TG-PTOX validated by isothermal titration calorimetry (ITC). For all panels, the top isotherm represents the heat of dissolution of compounds in solution, and the second isotherm represents the fragment binding titration.

To further elucidate the respective binding affinities, thermodynamic studies were conducted using isothermal titration calorimetry (ITC). Accurate binding affinity measurements using ITC with low *c*-values (ratio of macromolecule concentration to dissociation constant <1) can be achieved provided that (i) the binding enthalpy is sufficiently large (typically |Δ*H*| > 5 kcal·mol^−1^) to allow for the instrument sensitivity and (ii) a large proportion of the binding site (ideally >80%) is saturated with ligand at the end of the titration. Under the experimental conditions used, the latter should be true for dissociation constants around or below 5 mM. We were unable to reliably characterize the binding affinity for 4-MP-PTOX or 4-TG-PTOX to tubulin. Our ITC measurements had *K*_d_ values in the 0.5–0.4 mM range, with a Δ*G* of −3.4 – −3.1 kcal/mol, respectively (Fig. 7). Both compounds were found to be enthalpically-driven, with Δ*H* values of −7.4 and −5.3 kcal/mol, resulting in favorable binding entropy in all cases. The wide range of enthalpy values across the series likely reflect entropy/enthalpy compensation effects. The results indicate that the MP and TG fragments may enhance the affinity of 4-MP-PTOX and 4-TG-PTOX for binding to the tubulin by increasing the number of binding sites. However, it should be noted that Δ*H* values following data fitting of hyperbolic ITC titrations under low *c*-value conditions should be interpreted with caution. The binding stoichiometry cannot be measured under these conditions and it is not always possible to achieve complete saturation of the binding site at the end of the titration. The MP and TG fragments are postulated to bind in the GTP pocket. These two fragments showed an increase in affinity of 1.8 times and 12 times respectively, when titrated against tubulin. These results are consistent with a model where fragments bind tubulin in the GTP and colchicine pocket in a cooperative fashion. Lastly, both 4-MP-PTOX and 4-TG-PTOX binding to the tubulin complex showed significantly smaller binding enthalpies, resulting in favorable binding entropy.

In summary, we shed new light on two novel podophyllum tubulin inhibitors with promising anti-tumor therapy abilities. We substituted sulfur-containing heterocyclic compounds with a carbon-sulfur bond at position 4 of PTOX. Here we show that the two resultant compounds, 4-MP-PTOX and 4-TG-PTOX, greatly improve the therapeutic effect for microtubule damage in cancer cells by inhibiting tubulin polymerization, arresting mitosis, and inducing apoptosis. This study is a key step in the ongoing effort to develop better anti-tubulin compounds with enhanced anti-tumor capacities.

## Methods

### Computational docking simulations

Molecular docking of compounds into the X-ray structure of tubulin (PDB code: 1SA1) from the RCSB Protein Data Bank was carried out using the Auto-Dock software (version 4.2) via the graphic user interface Auto-Dock Tool Kit (ADT 1.5.4). The grid maps of docking studies were computed using AutoGrid 4, which is included in the Autodock4 software. The graphical user interface ADT was employed to set up the enzymes; in that, all hydrogen atoms were added, the Gasteiger charges were calculated, and nonpolar hydrogen atoms were merged with the carbon atoms. For macromolecules, the generated pdbqt files were saved. Then, a docking procedure based on the Auto-Dock software was applied to position the conformation of these compounds correctly with regard to their active sites. First, grid maps representing the tubulin protein in the actual docking process were constructed with Auto Grid. The points of the grids were thus set at 60 × 60 × 60 with a grid spacing of 0.375 Å to ensure that it was sufficiently large to include not only the active site but also significant portions of the surrounding surface.

### Chemicals

Standard PTOX (98%) was purchased from Shanxi Huisheng Medicament Technology Company, Ltd (Shanxi, China). 6-MP (98%) and 6-TG (98%) were purchased from Shanghai Jingchun Medicament Technology Company, Ltd (Shanghai, China). Precoated silica gel G plates for TLC were purchased from Merck Inc. (Darmstadt, Germany). Column chromatography was performed with Sephadex LH-20 gel (20-150 μm, Pharmacia & Upjohn Co., Switzerland). Flash column chromatography (FC) silica gel (SiO_2_; 200-300 meshes) was purchased from Qing Dao Haiyang Chemical Group Co. (Shandong, China). Methanol and acetonitrile were of high-performance liquid chromatography (HPLC) grade, and all other chemicals used for extraction and isolation were of analysis grade and commercially available. Deionized water was used throughout the study.

### Analytical method

The chemical reaction solution was alternately washed in deionized water and saturated NaHCO_3_. After removing the deionized water layer, drying the AcOEt layer by NaSO_4_, and removing AcOEt by rotary evaporation, the residue was ground by aether to obtain a white powder product. The powder product (2 mg) dissolved in 1 mL methanol/water (50:50 v/v) as pre-separated sample. Pre-separated sample was filtered (0.45-μm-micropore filter) and transferred into a sampling vial for HPLC analysis. The samples were filtered with a 0.45 μm micropore filter and transferred to a sampling vial for HPLC analysis. HPLC analysis was carried out on a Waters 600 Series HPLC system, equipped with 2487 UV detector. An Akasil C18 column (5 μm, 4.6 mm × 150 mm) was used. Mobile phase was methanol/water (50:50 v/v) and the pH was adjusted to 3.00 with formic acid. The HPLC oven temperature was maintained at 45 °C, and the detection wavelength was 230 nm or 219 nm. The flow rate was 0.8 ml/min. All ^1^H and ^13^C NMR spectra were recorded using a mercury-300BB spectrometer (Varian, USA). ESI-MS spectra were obtained with an Agilent MSD trap mass spectrometer. The separation was carried on a reversed-phase column with dimensions 150 × 4.6 mm and a particle size of 5 μm.

TLC analysis was carried out with a precoated silica gel G plate, and a small spot of solution containing the samples was applied onto the plate, approximately 1.5 cm from the bottom edge. The plate was then placed in a chamber with a solvent system consisting of chloroform/acetone (10:1 to 1:1, v/v). TLC spots were observed under an ultraviolet light (UV_254_) and then observed after spraying with H_2_SO_4_/MeOH (10:1 v/v) and heating to 110 °C.

### General procedure for synthesis of sulfur-substituted podophyllum derivatives

5 mL trifluoroacetic acid (TFA, 15 mL) were added dropwise to a mixture solution of PTOX (414 mg, 1 mmol) and 6-mercaptopurine (122 mg, 1 mmol) or 6-thioguanine (167 mg, 1 mmol). All reactions were stirred under nitrogen at 0 ^o^C for 48 h and monitored by TLC and then the liquid was dropped into 100 mL cold deionized water. Collect and dry the solid at 45 ^o^C after the mix was filtered and washed by deionized water (100 mL × 3). The solid was purified by chromatography on silica gel using CHCl_3_/ acetone 4:1 and 5:1 as eluant to give crude products, which were stable both at chemical purification stage and under assay conditions. Crude products were purified by Pre-HPLC to give pure product (purity: >95%), which was carried out on a Waters 600 Series HPLC system, equipped with 2487 UV detector. An Akasil C18 column (5 μm, 10 mm × 250 mm) was used. Mobile phase was methanol/water (45:55 v/v) and the pH was adjusted to 3.00 with formic acid. The HPLC oven temperature was maintained at 45 °C, and the detection wavelength was 230 nm or 219 nm. The flow rate was 1.5 ml/min.

After completion of the reaction, the sample was analyzed by TLC (eluted with CHCl_3_/acetone = 5/1); the reaction mixture was allowed to stand at room temperature for 1 h, and then the liquid was removed by rotary evaporation. The dry sample was re-dissolved in AcOEt (30 mL), and the solution was alternately washed in deionized water and saturated NaHCO_3_. After removing the deionized water layer, drying the AcOEt layer by NaSO_4_, and removing AcOEt by rotary evaporation, the residue was ground by aether to obtain a white powder product. The following two compounds were identified.

### 4β-*S*-(6-mercaptopurine-yl)sulfanyl-4-deoxy-podophyllotoxin (4-MP-PTOX) (1)

^1^H NMR (DMSO, 300 MHz) *δ* = 8.74 (s, 1H), 8.50 (s, 1H), 7.03 (s, 1H), 6.52 (s, 1H), 6.35 (s, 2H), 6.01 (s, 2H), 5.87 (d, ^2^*J* (4, 4) = 1.8 Hz, 1H), 4.62 (d, ^2^*J* (1, 1) = 3.0 Hz, 1H), 4.43–4.46 (m, 1H), 3.71–3.75 (m, 1H), 3.48–3.69 (m, 9H), 3.17–3.45 (m, 2H); ^13^C NMR (DMSO, 75 MHz) *δ* = 174.6 (COO*C*H_2_), 152.7 (COO), 152.0, 148.1 (C*C*H), 147.4 (C*C*H), 137.0 (CCH), 136.8 (CCH), 133.3 (CCH), 128.7 (CCH), 110.5 (CH), 110.2 (CH), 108.9 (CH), 102.1 (CH), 70.9 (CH), 60.6 (CH_2_), 56.5 (OCH_3_), 45.1 (CH), 43.7 (CH), 42.2 (CH), 37.3 (CH) ppm; EI MS: *m/z*: 549 [*M*^*+*^ *+* H], 571 [*M*^+^ + Na], 587 [*M*^+^ + K], HRMSEI (+) calcd for C_27_H_24_N_4_O_7_S, [M]^+^ 548.1153 found 548.5604.

### 4β-*S*-(6-thioguanine-yl)sulfanyl-4-deoxy-podophyllotoxin (4-TG-PTOX) (2)

^1^H NMR (methanol, 300 MHz) *δ* = 8.70 (s, 1H), 7.98 (s, 1H), 7.06 (s, 1H), 6.53 (s, 1H), 6.34 (s, 1H), 6.32 (s, 1H), 6.02 (s, 2H), 5.65 (s, 1H), 4.60–4.62 (d, ^2^*J* (1, 1) = 5.4 Hz, 1H), 3.62–3.40 (m, 1H), 3.30–3.43 (s, 2H); ^13^C NMR (methanol, 75 MHz) *δ* = 175.4 (COO*C*H_2_), 152.5 (COO), 148.7 (C*C*H), 147.8 (C*C*H), 147.7 (CO*C*H_3_), 135.1 (CCH), 133.8 (CCH), 131.5 (CCH), 128.5 (COH), 110.3 (CH), 108.8 (CH), 102.1 (CH), 71.2 (CH), 69.5 (CH_2_), 56.1 (OCH_3_), 55.5 (OCH_3_), 48.6 (CH), 48.5 (CH), 41.9 (CH), 38.5 (CH), 31.7 (CH) ppm; EI MS: *m/z*: 564.1 [*M*^*+*^ *+* H], 602.1 [*M*^+^ + K] , HRMSEI (+) calcd for C_27_H_25_N_5_O_7_S, [M]^+^ 563.1428 found 563.7245.

### Cytotoxicity Assay

Cytotoxicity assays were performed on the human gastric carcinoma cell line BGC-823, the human lung adenocarcinoma cell line A549, the human hepatocellular liver carcinoma HepG2, and the normal human hepatic immortal cell line HL-7702. Cells (3500–13,000) were seeded into 96-well microtest plates in 100 μL of culture medium per well (Falcon, CA). The cells were treated in triplicate with a gradient concentration of test compounds and incubated at 37 °C in RPMI-1640 with 100 mg L^−1^ kanamycin and 10% (v/v) fetal bovine serum in a humidified atmosphere containing 5% CO_2_ for 48 h. The plating efficiencies of the untreated cells varied between experiments with values ranging from 14 to 31% for the HL-7702 cells. For all cell lines, microculture tetrazolium [3-(4, 5-dimethylthiazol-2-yl)−2, 5-diphenyltetrazolium bromide, MTT; Sigma, St. Louis, MO] assay was performed to measure cytotoxic effects. Drug stock solutions were prepared in DMSO, and the final solvent concentration was ≤ 2% DMSO (v/v), a concentration that does not affect cell replication. The initial seeding densities varied among the cell lines to ensure a final absorbance reading in control (untreated) cultures within the range of 0.6–0.8 *A*_492_ units. Drug exposure was carried out for 2 days, and the IC_50_ value, i.e., the drug concentration that decreased the absorbance by 50%, was extrapolated from dose-response data. Each test was performed in triplicate, and the absorbance readings between the triplicates varied by no more than 5%.

### Statistical evaluation

Data presented as means ± SD. Statistical analyses were performed by the analysis of variance (ANOVA). All statistical analyses were performed using Origin version 8.0 (GraphPad Software, OriginLab Corp., Northampton, MA, USA). Sigmoidal dose responses and non-linear regression analyses were undertaken to identify half-maximal concentrations for each of the drugs. To evaluate differences in IC_50_ concentrations, analysis of variance combined with Tukey’s multiple range test was used.

### Immunofluorescence Staining of Tubulin

A549 tumour cells were maintained in four-well chamber slides (Lab-Tech) for 12 h prior to treatment with DMSO, 0.1 nM compound 2, 100 nM colchicine, 1.5 nM pacritaxel, or 500 nM doxorubicin for 24 h at 37 ^o^C. Cells were fixed with 4% paraformaldehyde in phospate buffered saline (PBS), permeabilized with 0.5% Triton X-100 in PBS, and then tubulin was immunostained with monoclonal antibody to tubulin (B5-1-2, Sigma) followed by fluorescein 5-isothiocyanate (FITC) conjugated secondary antibody. Nuclei were labeled with 4′, 6-diamidino-2-phenylindole (DAPI). Fluorescence labeled tubulin and nuclei were observed using a Zeiss Axioplan fluorescence microscope, and images were captured by a XL16 Excel cooled digital camera controlled by the Dage Exponent software (Dage-MTI). Final images were prepared using Adobe Photoshop. The cells were then processed for immunofluoscence staining and confocal microscopy (× 600).

### Tubulin Assembly

Purification of calf brain tubulin and chemicals were followed as previously described by Andreu^20^. Ligands were dissolved in DMSO at 20 mM and kept at −80 °C. Work solutions were done in DMSO and kept at −20 °C. The 50% inhibitory ligand concentration of tubulin assembly was determined with a centrifugation assay. Tubulin was equilibrated prior to use in 3.4 M glycerol, 1 mM EGTA, 0.1 mM GTP, pH 7.0, buffer through a 25 cm × 0.9 cm Sephadex G-25 column. Aggregates were removed by a centrifugation at 90000 g × 10 min in a TLA 120 rotor at 4 °C in an Optima TLX centrifuge. Tubulin concentration was determined as described by Andreu^21^. Tubulin was kept at 4 °C, and 0.9 mM GTP and 6 mM MgCl_2_ were added to the sample. The solution was distributed in 200 μL polycarbonate tubes for the TL100 rotor. Growing concentrations of the ligands ranging from 0 to 25 μM were added to the samples (DMSO content of the samples, 2.5%), which were incubated for 30 min at 37 °C. Microtubules were separated from unassembled tubulin by a centrifugation at 90000 g × 10 min in a TLA100 rotor at 37 °C in an Optima TLX centrifuge. The supernatant containing unassembled tubulin was carefully collected and the microtubule pellet resuspended in 10 mM sodium phosphate buffer, pH 7.0, containing 1% SDS. Both supernatants and pellets were diluted 1:5 in the same buffer, and tubulin concentrations were measured fluorometrically (λexc = 280; λems = 323) using tubulin standards calibrated spectrophotometrically. The 50% inhibitory ligand concentration of tubulin assembly was determined with a centrifugation assay that measured the decrease in the concentrations of microtubules assembled in the presence of different concentrations of the compound.

### Surface plasmon resonance-based binding assays

Based on the surface plasmon resonance (SPR) technology, the binding affinity of compounds to tubulin was determined with the ProteOn^TM^ XPR36 Protein Interaction Array System (Bio-Rad, Hercules, CA). For this purpose, tubulin was dissolved in 10 mM sodium acetate buffer (pH 3.5) and immobilized to the ProteOn^TM^ GLH sensor chip with the ProteOn^TM^ amine coupling kit. The final immobilization level was 8000 RU (Response unit). Compounds 4-MP-PTOX and 4-TG-PTOX was two-fold diluted from 40 μM to 2.5 μM in HBS-T buffer (10 mM HEPES, 150 mM NaCl, 3.4 mM EDTA, 0.005% Tween 20, pH 7.4). Compounds 4-MP-PTOX and 4-TG-PTOX of different concentrations was then injected at a flow rate of 30 μl/min for 200 s, which was followed by a 300-s dissociation phase. Data were analyzed with the ProteOn^TM^ Manager software, fitted to the 1:1 Langmuir model.

### Cell cycle analysis

Cell cycle arrest detection in A549 cells using propidium iodide (PI) double staining treatment with different concentrations of 4-TG-PTOX (i.e., 0.1, 1, and 5 μM) for 6, 12, 24, 48 h, respectively. The A549 cell line was used for cell cycle analysis. Cells were treated in triplicate with a gradient concentration of test compounds and incubated at 37 °C in RPMI-1640 with 100 mg L^−1^ kanamycin and 10% (v/v) fetal bovine serum in a humidified atmosphere containing 5% CO_2_ for 48 h. Untreated and drug-treated cells were centrifuged and fixed overnight in 70% ethanol at 4 °C. They were then washed three times with PBS, incubated for 1 h with 1 mg/ mL RNase A and 20 mg/ mL propidium iodide at room temperature, and analyzed with a FACSCalibur flow cytometer (BD, USA).

### Cell apoptosis analysis

The apoptosis ratios induced by compounds caused apoptosis in tumour cells was quantitatively assessed by a FACSCalibur flow cytometer (BD, USA). Cell apoptosis-inducing detection in A549 cells treatment with different concentrations of 4-TG-PTOX (i.e., 0.1, 1, and 5 μM) for 6, 12, 24, 48 h, respectively. In the early stages of apoptosis, phosphatidylserine (PS) was translocated from the inside of the cell membrane to the outside. Annexin V, a calcium dependent phospholipid-binding protein associated with a high affinity for phosphatidylserine, was used to detect early apoptotic cells. Propidine Iodide (PI) was a red fluorescent dye and stained cells that had lost membrane integrity. So, cells stained with FITC-annexin V and PI were discriminated necrotic cells (Q1, Annexin-/PI +), late apoptotic cells (Q2, Annexin +/PI+), intact cells (Q3, Annexin -/PI -) and early apoptotic cells (Q4, Annexin+/PI -).

### Isothermal Titration Calorimetry (ITC)

ITC experiments were performed using a TAM III instruments (Q Series™ Thermal Analysis). Fragments were solubilized in either 5% or 2% vol/vol DMSO, at a range of concentration of 10-20 mM. Protein solutions were dialyzed overnight in HBS-T buffer. The thermodynamic characterization and affinity determined through the use of the Origin software by using a single ligand binding model.

## Author Contributions

W.Z. and Y.J.T. conceived the project. W.Z. designed the experiments, W.Z. and J.K.B. implemented the analysis workflow and conducted the experiments. W.Z., J.K.B., H.M.L., and T.C. analyzed and interpreted the results, W.Z. prepared all figures and tables, W.Z. and Y.J.T. wrote the manuscript. All authors reviewed, commented on, and approved the final manuscript.

## Additional Information

**How to cite this article**: Zhao, W. *et al*. Tubulin structure-based drug design for the development of novel 4ß-sulfur-substituted podophyllum tubulin inhibitors with anti-tumor activity. *Sci. Rep.* **5**, 10172 doi: 10.1038/srep10172 (2015).

## Supplementary Material

Supplementary Information

## Figures and Tables

**Table 1:** IC_50_ values of the compounds against tumor cells and normal human cells.

	**Cytotoxic activity (IC**_**50**_**, μM)**^a^			
**Compound**	**BGC-823**^b^	**A549**^b^	**HepG2**^b^	**HL-7720**^b^
4-MP-PTOX	8.72 ± 3.15	1.12 ± 0.35	1.14 ± 1.07	11.30 ± 0.57
4-TG-PTOX	4.40 ± 1.55	0.57 ± 0.92	0.30 ± 0.34	13.51 ± 2.41
PTOX	18.32 ± 2.22	20.15 ± 2.75	8.36 ± 1.42	4.31 ± 1.46
Colchicines	12.17 ± 1.26	12.23 ± 0.76	13.22 ± 0.88	6.42 ± 1.03
Nocodazole	38.89 ± 3.12	27.44 ± 2.92	18.35 ± 3.71	8.51 ± 1.73

^a^Standard deviation (SD) of triplicate samples was calculated from three independent samples, mean ± SD (n = 3)

^b^MTT methods, drug exposure was for 48 h.

## References

[b1] Pennisi E. Structure of key cytoskeletal protein tubulin revealed. Science . 279, 176–177 (1998). 9446222 10.1126/science.279.5348.176

[b2] Dumontet C. & Jordan M. A. Microtubule-binding agents: a dynamic field of cancer therapeutics. Nat. Rev. Drug Discov. 9, 790–803 (2010). 20885410 10.1038/nrd3253 PMC3194401

[b3] Voigt T. C. *et al.* A natural product inspired tetrahydropyran collection yields mitosis modulators that synergistically target CSE1L and tubulin. Angew. Chem. Int. Ed. , 52, 410–414 (2013). 10.1002/anie.201205728 23080551

[b4] Schönbrunn E. *et al.* Crystallization of a macromolecular ring assembly of tubulin liganded with the anti-mitotic drug podophyllotoxin. J. Struct. Biol. 128, 211–215 (1999). 10600574 10.1006/jsbi.1999.4183

[b5] Alam M. A. & Naik P. K. Applying linear interaction energy method for binding affinity calculations of podophyllotoxin analogues with tubulin using continuum solvent model and prediction of cytotoxic activity J. Mol. Graph. Model , 27, 930–943 (2009). 19286405 10.1016/j.jmgm.2009.02.003

[b6] Hall L. H. Bioisosterism: quantitation of structure and property effects. Chem. Biodivers , 1, 138–151 (2004). 17191782 10.1002/cbdv.200490006

[b7] Bai J. K. Zhao W. Li H. M. Tang Y. J. Novel biotransformation process of podophyllotoxin to 4’-sulfur-substituted podophyllum derivates with anti-tumor activity by *penicillium purpurogenum* Y.J. Tang. Curr. Med. Chem. 19, 927–936 (2012). 22214458 10.2174/092986712799034914

[b8] Satchi M. *et al.* Is treatment with 6-mercaptopurine for colitis associated with the development of colorectal cancer? Inflamm. Bowel. Dis. 19, 785–788 (2013). 23392347 10.1097/MIB.0b013e318289664c

[b9] Martínez-Fernández L. González L. & Corral, I. An ab initio mechanism for efficient population of triplet states in cytotoxic sulfur substituted DNA bases: the case of 6-thioguaninew Chem. Commun. 48, 2134–2136 (2012). 10.1039/c2cc15775f 22245861

[b10] Ravelli R. B. G. *et al.* Tubulin-podophyllotoxin: stathmin-like domain complex. Nature , 428, 198–202 (2004). 15014504 10.1038/nature02393

[b11] Abad A. *et al.* Synthesis and antimitotic and tubulin interaction profiles of novel pinacol derivatives of podophyllotoxins J. Med. Chem. 55, 6724–6737 (2012). 22607205 10.1021/jm2017573

[b12] Joshi H. C. Palacios M. J. Mcnamara L. Cleveland D. W. Tubulin is a centrosomal protein required for cell cycle-dependent microtubule nucleation. Nature , 356, 80–83 (1992). 1538786 10.1038/356080a0

[b13] Sacket D. L. Podophyllotoxin, steganacin and combretastatin: natural products that bind at the colchicine site of tubulin. Pharmac. Ther. 59, 163–228 (1993). 10.1016/0163-7258(93)90044-e 8278462

[b14] Hanahan D. Weinberg R. A. Hallmarks of Cancer: The Next Generation. Cell 144, 646–674 (2011). 21376230 10.1016/j.cell.2011.02.013

[b15] Wallace B. D. *et al.* Alleviating cancer drug toxicity by inhibiting a bacterial enzyme. Science 330, 831- (2010). 21051639 10.1126/science.1191175 PMC3110694

[b16] Huang H. S. *et al.* Topoisomerase inhibitors unsilence the dormant allele of Ube3a in neurons. Nature 481, 185–191 (2012). 10.1038/nature10726 PMC3257422 22190039

[b17] Marsilje T. H. *et al.* Synthesis, Structure−Activity Relationships, and in Vivo Efficacy of the Novel Potent and Selective Anaplastic Lymphoma Kinase (ALK) Inhibitor 5-Chloro-N2-(2-isopropoxy-5-methyl-4-(piperidin-4-yl)phenyl)-N4-(2-(isopropylsulfonyl)phenyl)pyrimidine-2,4-diamine (LDK378) Currently in Phase 1 and Phase 2 Clinical Trials. J. Med. Chem. 56, 5675–5690 (2013). 23742252 10.1021/jm400402q

[b18] Riva E. *et al.* Synthesis and biological evaluation of new camptothecin derivatives obtained by modification of position 20. Bioorg. Med. Chem. 18, 8660–8668 (2010). 21071230 10.1016/j.bmc.2010.09.069

[b19] Warrener R. *et al.* Tumor cell-selective cytotoxicity by targeting cell cycle checkpoints. FASEB. J. 17, 1550–1552 (2003). 12824307 10.1096/fj.02-1003fje

[b20] Diaz J. F. Andreu J. M. Assembly of purified GDP-tubulin into microtubules induced by Taxol and Taxotere: reversibility, ligand stoichiometry, and competition. Biochemistry , 32, 2747–2755 (1993). 8096151 10.1021/bi00062a003

[b21] Andreu J. M. Gorbunoff M. J. Lee J. C. Timasheff S. N. Interaction of tubulin with bifunctional colchicine analogues: an equilibrium study. Biochemistry , 23, 1742–1752 (1984). 6722122 10.1021/bi00303a025

