# Supplementary material for: Tubulin structure-based drug design for the development of novel 4β-sulfur-substituted podophyllum tubulin inhibitors with anti-tumor activity
Source: Sci Rep. 2015 May 11;5:10172. doi: 10.1038/srep10172 (PMC4426677; doi:10.1038/srep10172)
Supplement: Supplementary Information [file srep10172-s1.doc]

**Tubulin structure-based drug design for the development of novel 4β-sulfur-substituted podophyllum tubulin inhibitors with anti-tumor activity**

**Wei Zhao *^a^*,** **Jia-Ke Bai** ***^a^*, Hong-Mei Li *^a^*, Tao Chen *^b^* and Ya-Jie Tang *^a^****

***^a^*** *Key Laboratory of Fermentation Engineering (Ministry of Education), Hubei Provincial Cooperative Innovation Center of Industrial Fermentation, Hubei University of Technology, Wuhan 430068, China*

***^b^*** *Key Laboratory of Systems Bioengineering (Ministry of Education),* *School of Chemical Engineering and Technology, Tianjin University, Tianjin* *300072, China*

*Corresponding author. Tel. & Fax: +86-27-5975.0491 Email: yajietang@hotmail.com

**
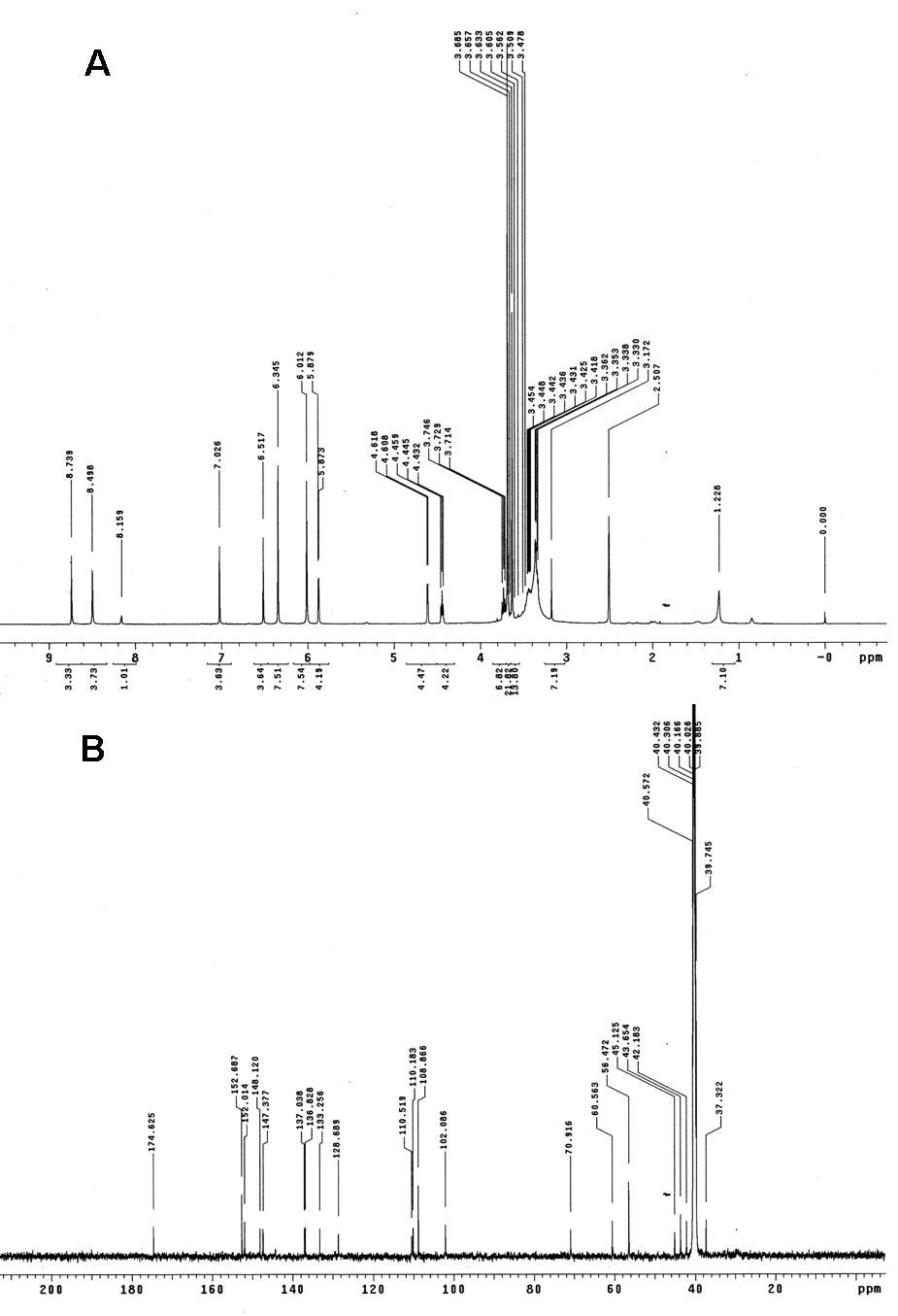
**

**Figure 1S.** ^1^H NMR spectra of 4β-*S*-(6-mercaptopurine-yl)sulfanyl-4-deoxy-podophyllotoxin (A) and ^13^C NMR spectra of 4β-*S*-(6-mercaptopurine-yl)sulfanyl-4-deoxy-podophyllotoxin (B)


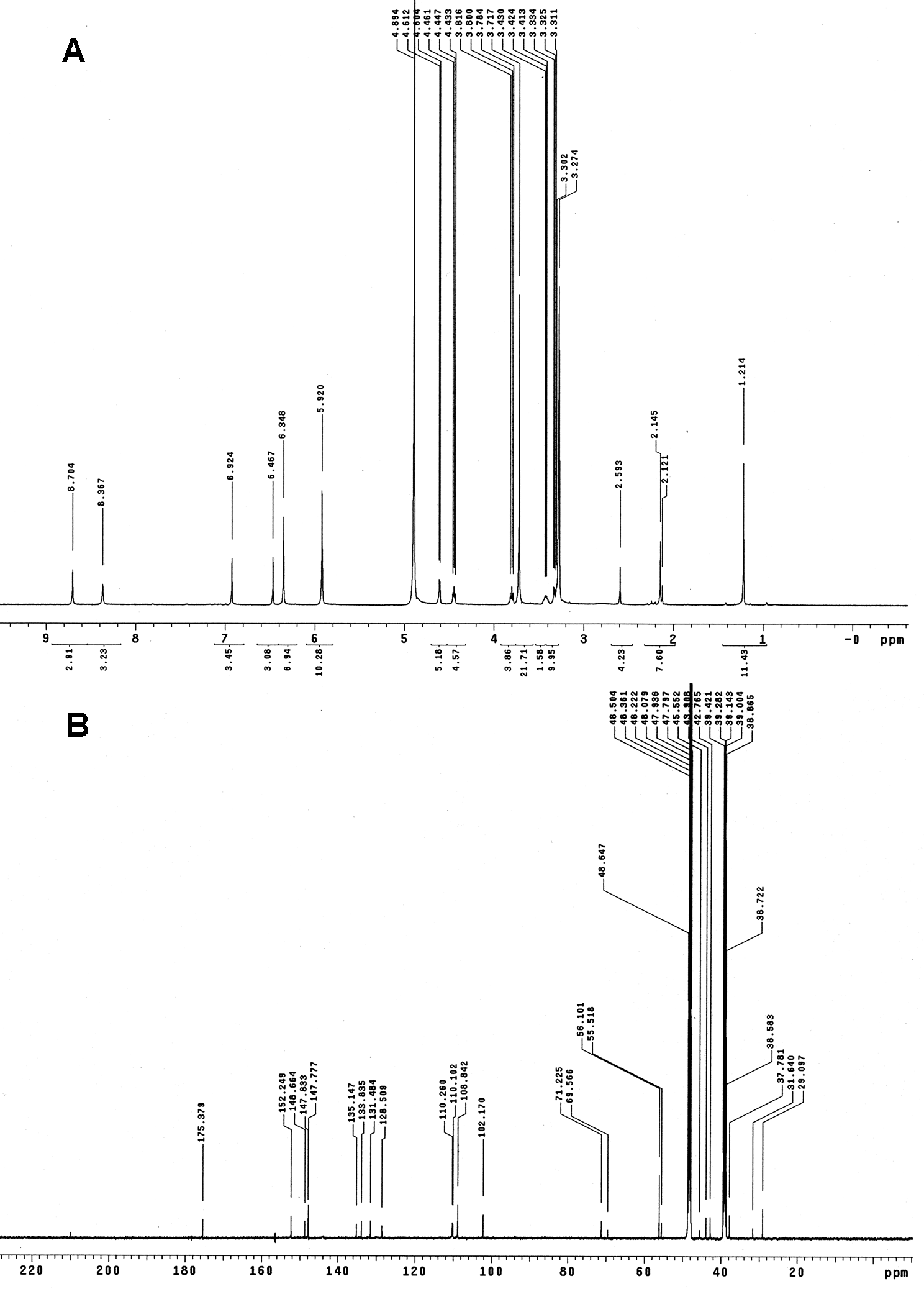


**Figure 2S.** ^1^H NMR spectra of 4β-*S*-(6-thioguanine-yl)sulfanyl-4-deoxy-podophyllotoxin (A) and ^13^C NMR spectra of 4β-*S*-(6-thioguanine-yl)sulfanyl-4-deoxy-podophyllotoxin (B)


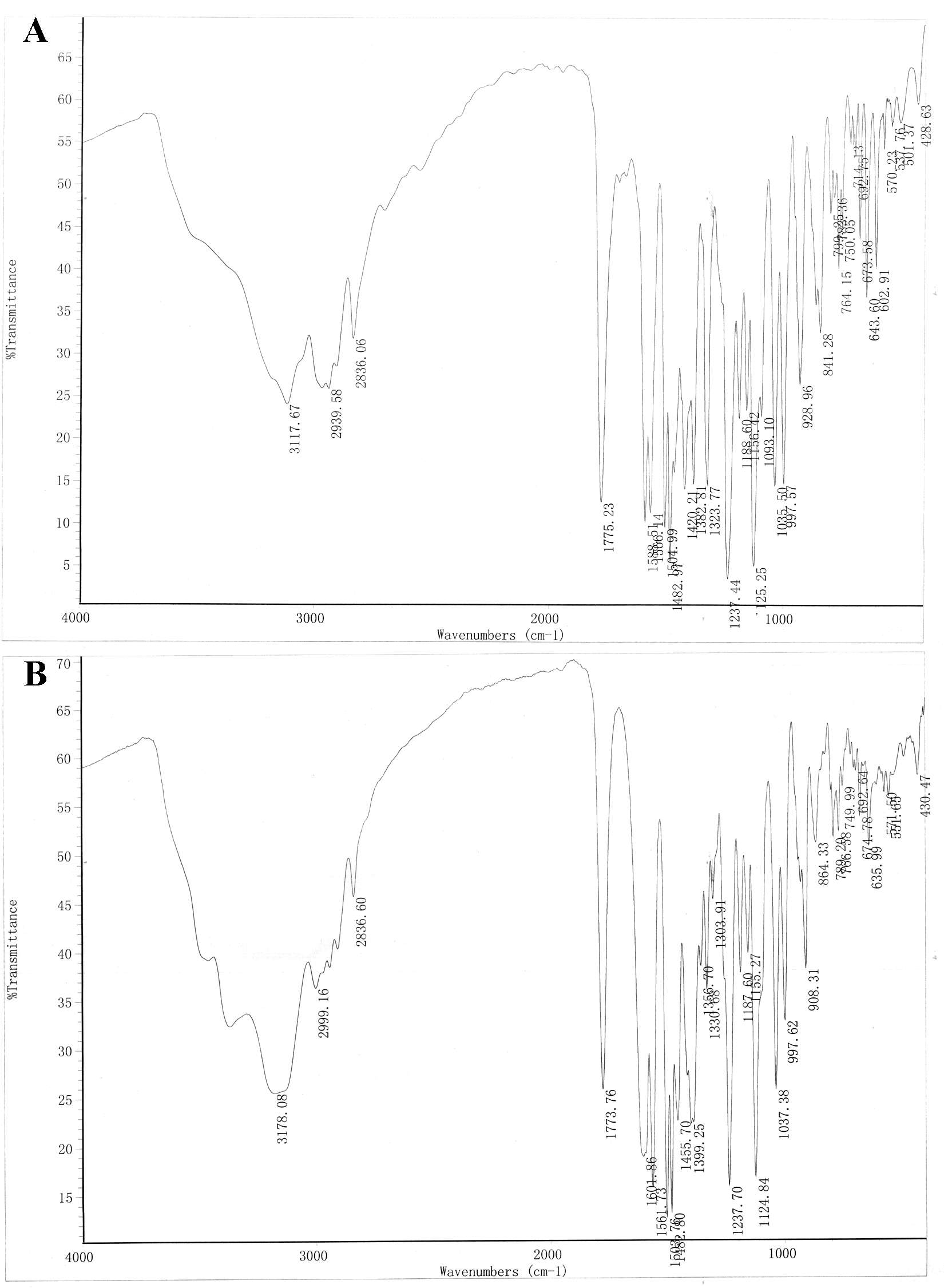


**Figure 3S.** FT-IR spectrum of products. A. Spectrum of 4β-S-(6-mercaptopurine)-4-deoxy-podophyllotoxin (4-MP-PTOX). B. Spectrum of 4β-S-(6-thioguanine)-4-deoxy-podophyllotoxin (4-TG-PTOX).


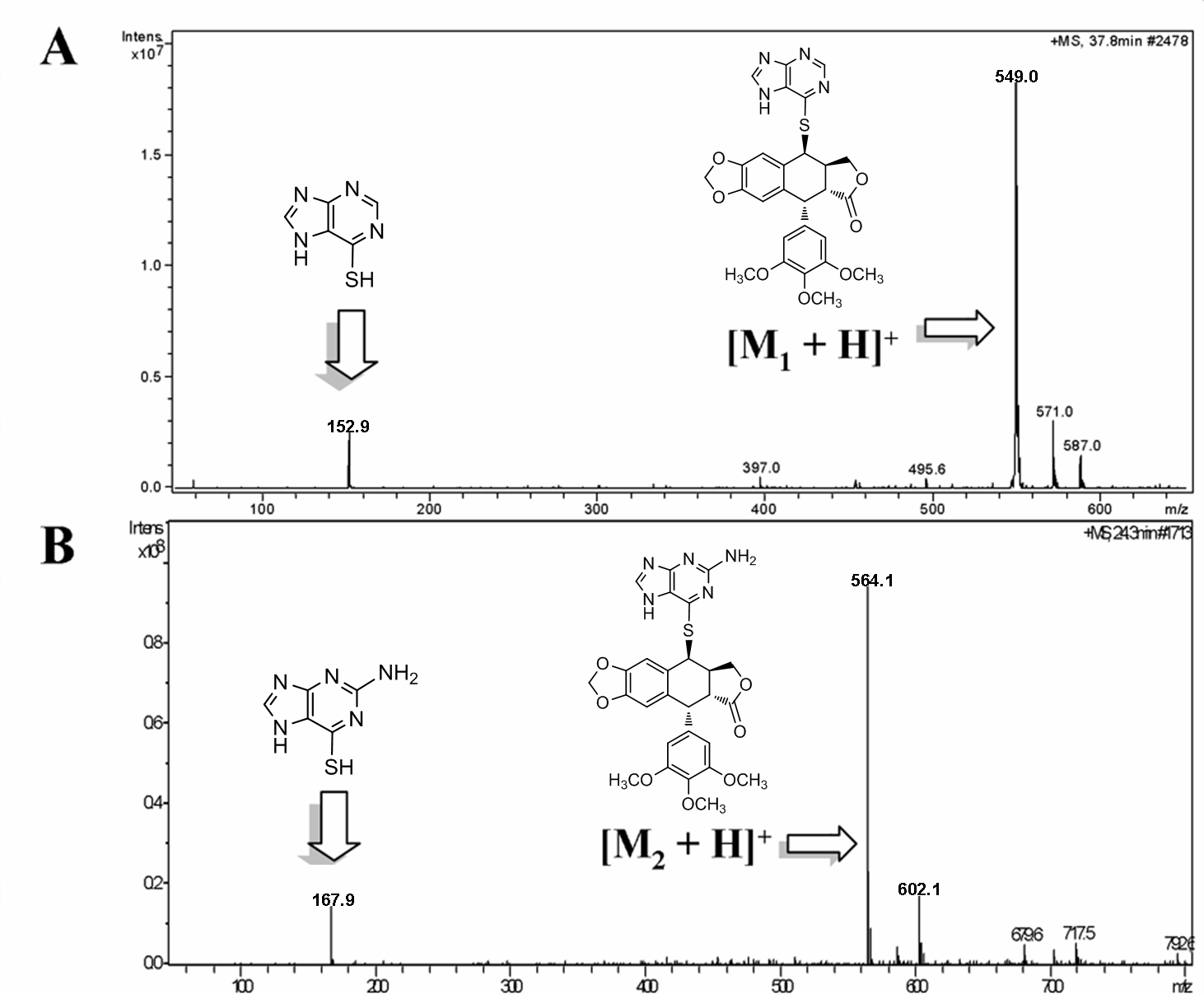


**Figure 4S.** HPLC-ESI-MS spectrum of products. A. Spectrum of 4β-S-(6-mercaptopurine)-4-deoxy-podophyllotoxin (4-MP-PTOX). B. Spectrum of 4β-S-(6-thioguanine)-4-deoxy-podophyllotoxin (4-TG-PTOX). The insets show the respective HPLC-MS ionization.
